# Expression of PD-L1 in triple-negative breast cancer based on different immunohistochemical antibodies

**DOI:** 10.1186/s12967-016-0925-6

**Published:** 2016-06-10

**Authors:** Woo Young Sun, Yu Kyung Lee, Ja Seung Koo

**Affiliations:** Department of Surgery, Daejeon St. Mary’s Hospital, The Catholic University of Korea College of Medicine, Seoul, South Korea; Department of Pathology, Yonsei University College of Medicine, Severance Hospital, 50 Yonsei-ro, Seodaemun-gu, Seoul, 120-752 South Korea

**Keywords:** Breast cancer, PD-L1, Monoclonal antibody, Immunohistochemistry, Triple negative

## Abstract

**Background:**

To date, there are no effective therapeutic targeting agents for triple-negative breast cancer (TNBC), and PD-L1 has presented potential as an effective marker of immunotherapeutic agents. The aim of this study was to evaluate the expression of PD-L1 by three different immunohistochemical antibodies in TNBC.

**Methods:**

Interpretation of all three PD-L1 antibodies showed good concordance among three readers (kappa value >0.610) in both cancer cells and immune cells. Using a tissue microarray (TMA) constructed from 218 cases of TNBC, we performed immunohistochemical staining using three of the most popular commercially used PD-L1 monoclonal antibodies (clones 28-8, E1L3N and SP142) in cancer cells and immune cells.

**Results:**

Using various cut-off values of previous studies (1, 5, 10 and 50 %), the expression rates in cancer cells were: PD-L1 (E1L3N) (14.7, 14.7, 11.0, 2.3 %), PD-L1 (28-8) (13.3, 12.4, 10.1, 1.8 %), and PD-L1 (SP142) (11.5, 11.0, 6.9, 0.5 %), respectively. At the 5 % cut-off value, the discordance rate among the three antibodies was 6.0–10.6 % and was highest between PD-L1 (SP142) and the other two antibodies. The expression rates in immune cells were PD-L1 (E1L3N) (37.6 %), PD-L1 (28-8) (36.7 %), and PD-L1 (SP142) (19.3 %), and the discordance rate among the three antibodies ranged from 13.8 to 24.8 % and was also highest between PD-L1 (SP142) and the other two antibodies. Among stromal histologic types, higher PD-L1 expression in cancer cells and immune cells was measured in inflammatory-type (p < 0.05). The absence of PD-L1 (28-8) staining in immune cells was associated with shorter disease free survival (DFS) and overall survival (OS) (p = 0.043, and p = 0.021) in univariate analyses, and with shorter OS in multivariate Cox analysis (hazard ratio: 5.429, 95 % CI 1.214–24.28, p = 0.027).

**Conclusions:**

PD-L1 detection in cancer cells and immune cells varied by antibody clone. The greatest amount of staining occurred with PD-L1 (E1L3N), followed by PD-L1 (28-8) and PD-L1 (SP142). The concordance rate among monoclonal PD-L1 antibodies was higher between PD-L1 (28-8) and PD-L1 (E1L3N). To determine the gold standard antibody and the most appropriate cut-off value, further study of the clinical trial group treated with PD-L1 inhibitor is necessary.

**Electronic supplementary material:**

The online version of this article (doi:10.1186/s12967-016-0925-6) contains supplementary material, which is available to authorized users.

## Background

Breast cancer is a heterogeneous disease that has several clinical, histological, and genetic forms. There have been many attempts to categorize this heterogeneous disease, and now molecular classification into five groups (luminal A, luminal B, HER-2, normal breast-like, and basal-like) can be accomplished by gene-expression profiling [1, 2].

Among these molecular subtypes, basal-like breast cancer has a more aggressive clinical course than other subtypes and is commonly known as triple-negative breast cancer (TNBC), which is defined clinically as lacking estrogen receptor (ER) and progesterone receptor (PR) and human epidermal growth factor receptor 2 (HER2). TNBC accounts for 10–17 % of all breast cancers [3–8]. Highly heterogeneous and known to have several molecular subtypes, TNBC is difficult to treat because it does not respond to hormonal or targeted therapy such as Herceptin, except chemotherapy [9–11].

Programmed death 1 (PD-1) is a check point molecule in immune reactions and can be expressed in various immune cells [12]. PD-L1, a ligand of PD-1, is expressed in cancer cells and the binding of PD-L1 with PD-1 helps cancer cells avoid antitumor immune responses [13, 14]. PD-L1 expression has been reported at rates of 20–70 % in lung cancer [13, 15–18], urinary bladder cancer [19], malignant melanoma [20], and ovarian cancer [21]. PD-L1 was expressed in not only tumor cells but immune cells in previous studies of breast cancer [22–24], and lung cancer [25]. In addition, its expression in immune cells demonstrated clinical implication [23]. Therefore, the evaluation of PD-L1 should be performed in both cancer cell and immune cells.

Target therapy for PD-L1 in PD-L1-expressing cancers represents a possible treatment for inducing antitumor immune responses. PD-L1-targeted therapy has been investigated in preclinical and clinical trials in many tumors [15–17, 19, 26–28] and anti-PD-L1 antibodies such as BMS-936,559 [29] and MPDL3280A [16, 19] have been developed. Knowledge of the expression of PD-L1 in cancer cells plays an important role in tailored therapy planning, and is easily evaluated using immunohistochemistry (IHC) with a monoclonal PD-L1 antibody in formalin-fixed and paraffin-embedded (FFPE) specimens. Multiple monoclonal PD-L1 antibodies such clone 28-8 [30], clone 22C3 [31], clone SP142 [16, 19], and clone E1L3N [32, 33] have been commercially developed.

The aim of this study was to evaluate the expression of PD-L1 using different immunohistochemical antibodies in TNBC and associated clinical implications.

## Methods

### Patient selection

We analyzed 218 patients with TNBC who underwent surgery at Severance Hospital between January 2000 and December 2006. This study was approved by the Institutional Review Board of Severance Hospital. All patients were diagnosed as having invasive ductal carcinoma, not otherwise specified (NOS) by pathologists. We defined TNBC as when IHC for ER, PR, and HER-2 and FISH for HER-2 were all negative.

ER and PR immunohistochemistry signal were considered positive when more than 1 % of invasive tumor cells were expressed [34]. HER-2 staining was scored according to the American Society of Clinical Oncology (ASCO)/College of American Pathologists (CAP) guideline using the following categories: 0, no immunostaining; 1+, weak incomplete membranous staining in any proportion of tumor cells; 2+, complete membranous staining, either non uniform or weak in at least 10 % of tumor cells; and 3+, uniform intense membranous staining in >30 % of tumor cells [35]. Cases with 0 to 1+ were regarded as negative and case with 3+ was considered as positive. Cases with HER-2 2+ were investigated with FISH (Vysis pathvision HER-2 kit) for HER-2 gene status. As proposed by the ASCO/CAP guideline, an absolute HER-2 gene copy number lower than four or HER-2 gene/chromosome 17 copy number ratio (HER-2/Chr17 ratio) of less than 1.8 was considered HER-2 negative; an absolute HER-2 copy number between 4 and 6 or HER-2/Chr17 ratio between 1.8 and 2.2 was considered HER-2 equivocal; and an absolute HER2 copy number greater than 6 or HER-2/Chr17 ratio higher than 2.2 was considered HER-2 positive.

Formalin-fixed and paraffin-embedded tissue specimens from 218 cases of primary breast cancer were included. All archival hematoxylin and eosin (H&E)-stained slides for each case were reviewed by one pathologists (Koo JS). The histological grade was accessed using Nottingham grading system [36]. According to the microscopic cancer stoma findings, TNBC was categorized as follows [37]: *desmoplastic type*, with cellular fibroblast/myofibroblast proliferation as the main stromal content; *sclerotic type*, which has a small cell component and fibrotic collagenous component as its main stromal content; or *inflammatory type*, with stroma mainly composed of inflammatory cells such as lymphocytes. Tumor staging was based on the 8th American Joint Committee on Cancer (AJCC) criteria. Disease-free survival (DFS) time was measured from the date of the first curative surgery to the date of the first locoregional or systemic relapse, or death without any type of relapse. Overall survival (OS) time was calculated from the date of the first curative operation to the date of the last follow-up or death from any cause. Histologic parameters were evaluated from the H&E-stained slides. Clinical parameters evaluated in each tumor included patient age at initial diagnosis, lymph node status, local recurrence, systemic recurrence, and patient’s survival.

### Tissue microarray

On H&E-stained slides of tumors, a representative area was selected and a corresponding spot was marked on the surface of the paraffin block. Using a punch machine, the selected area was punched out and a 3-mm tissue core was placed into a 6 × 5 recipient block. More than two tissue cores were extracted to minimize extraction bias. Each tissue core was assigned with a unique tissue microarray location number that was linked to a database containing other clinicopathologic data.

### Immunohistochemistry (IHC)

The antibodies and dilution used for IHC are shown in Additional file 1: Table S1. All immunohistochemistry was performed with formalin-fixed, paraffin-embedded tissue sections using an automatic immunohistochemistry staining device (Benchmark XT, Ventana Medical System, Tucson, AZ, USA). Briefly, 5-µm-thick formaldehyde fixed paraffin-embedded tissue sections were transferred onto adhesive slides and dried at 62 °C for 30 min. Standard heat epitope retrieval was performed for 30 min in ethylene diamine tetraacetic acid, pH 8.0, in the autostainer. The samples were then incubated with primary antibodies. After incubation with primary antibodies, The sections were subsequently incubated with biotinylated anti-mouse immunoglobulins, peroxidase-labeled streptavidin (LSAB kit, DakoCytomation), and 3,30-diaminobenzidine. Negative control samples were processed without the primary antibody. Slides were counterstained with Harris hematoxylin. Positive control tissue was used as per the manufacturer’s recommendation (placenta and tonsil). Slides were counterstained with Harris hematoxylin. Optimal primary antibody incubation times and concentrations were determined by serial dilution for each immunohistochemical assay using a tissue block fixed and embedded exactly as for the experiments.

### Interpretation of immunohistochemical staining

We evaluated the expression of immunohistochemical markers in cancer cells and peri-tumoral immune cells by light microscopy. Expression in cancer cells was analyzed by various cut-off values (1, 5, 10 and 50 %) and expression in immune cells was analyzed as follows: negative, no immunostaining; low-positive, stained immune cells ≤30/high power field (HPF); and high-positive, stained immune cells >30/HPF.

Interpretation of immunohistochemical staining was performed independently by three researchers (SWY, LYK, KJS). We investigated the proportion of expression (%) in cancer cells according to the above criteria, counted the number of expressed immune cells. Each of three pathologists analyzed the samples and reached a conclusion, which were then analyzed to determine inter-reader concordance. The final results were determined through discussion and multi-view microscopy in the cases that showed discrepancy among the three pathologists.

### Statistical analysis

Data were analyzed using SPSS for Windows, Version 12.0 (SPSS Inc., Chicago, IL, USA). For determination of statistical significance, Student’s *t* and Fisher’s exact tests were used for continuous and categorical variables, respectively. The inter-reader concordance rate and concordance rate among PD-L1 antibodies were analyzed using the Kappa-Cohen method. Statistical significance was when p < 0.05. Kaplan–Meier survival curves and log-rank statistics were employed to evaluate time to tumor recurrence and overall survival. Multivariate regression analysis was performed using Cox proportional hazards model.

## Results

### Basal characteristics of TNBC

Among the 218 TNBC patients, a desmoplastic type was observed in 138 cases (63.3 %), inflammatory type in 63 cases (28.9 %), and sclerotic type in 17 cases (7.8 %). There was no difference in clinicopathologic factors according to stromal type (Additional file 1: Table S2).

### Inter-reader reproducibility for monoclonal PD-L1 antibodies

The kappa values of all three PD-L1 antibodies were >0.610 in both cancer cells and immune cells. In cancer cells, the concordance rate was highest when using a 1 % cut-off value, while the lowest concordance rate was seen at the 10 % cut-off value (Table 1).

**Table 1:** Kappa value for inter-reader reproducibility of PD-L1 monoclonal antibodies

Reader	PD-L1 (28-8)					PD-L1 (E1L3N)					PD-L1 (SP142)				
	Tumor cell				Immune cell	Tumor cell				Immune cell	Tumor cell				Immune cell
	1 %	5 %	10 %	50 %		1 %	5 %	10 %	50 %		1 %	5 %	10 %	50 %	
#1 to #2	1.000	0.913	0.770	0.838	0.903	1.000	0.943	0.876	0.887	0.914	1.000	0.871	0.889	1.000	0.928
#1 to #3	1.000	0.958	0.923	0.838	0.894	1.000	0.981	0.820	0.887	0.888	1.000	0.976	0.924	1.000	0.858
#2 to #3	1.000	0.913	0.794	0.628	0.798	1.000	0.923	0.670	1.000	0.870	0.977	0.842	0.803	1.000	0.788

### PD-L1 monoclonal antibody staining in TNBC cells and immune cells

**Fig. 1:**
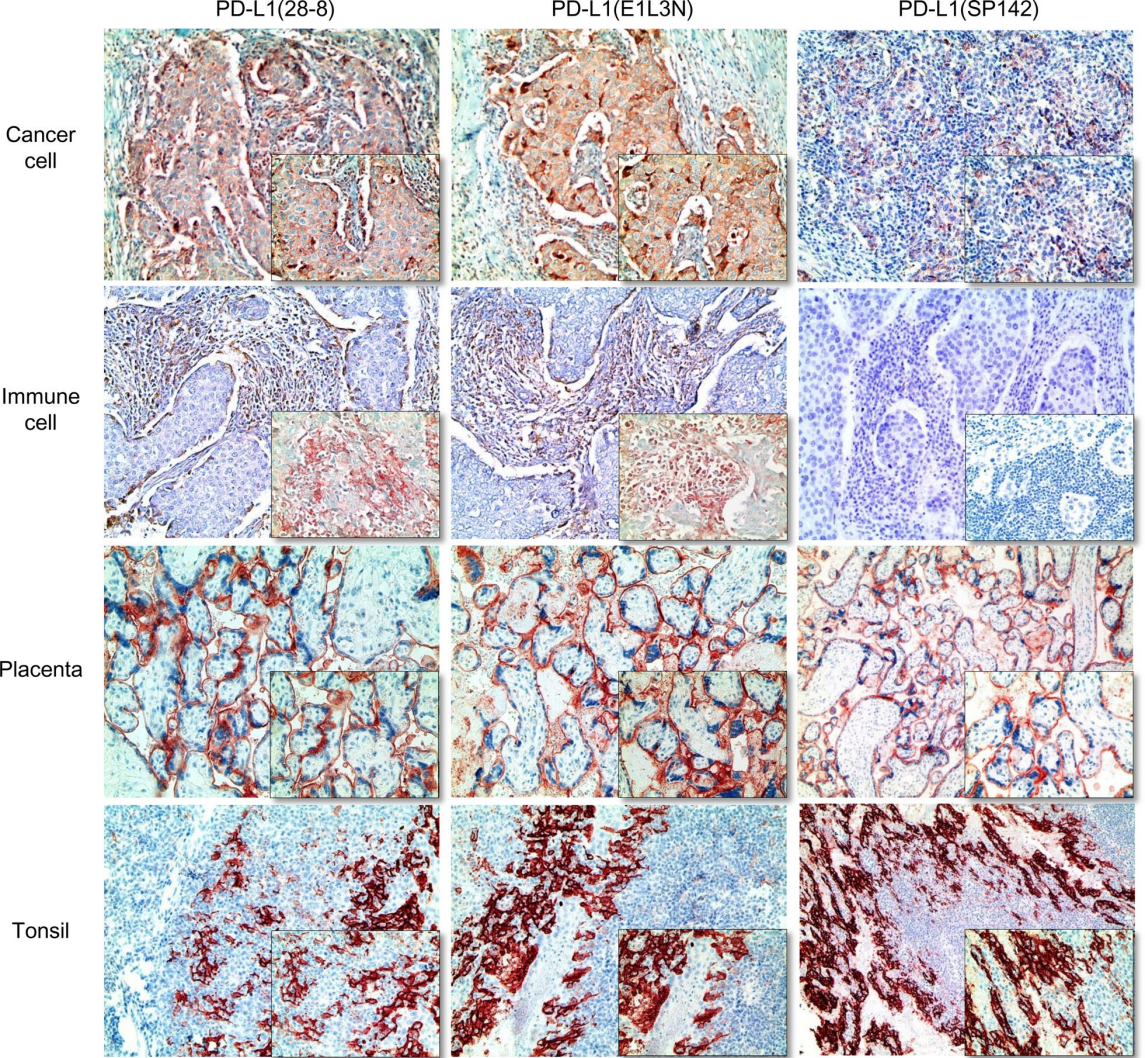
Staining with PD-L1 monoclonal antibodies in TNBC. PD-L1 expression in cancer cells was similarly positive for PD-L1 (clone 28-8) and PD-L1 (clone E1L3N) antibodies, but low for PD-L1 (clone SP142). Both PD-L1 (clone 28-8) and PD-L1 (clone E1L3N) stained positive in many immune cells, while PD-L1 (clone SP142) was mostly negative in this cell type. Placenta and tonsil tissue were used as the positive control

Among the different PD-L1 monoclonal antibodies, PD-L1 (E1L3N) showed the highest expression rate in cancer cells (14.7, 14.7, 11.0, 2.3 %) and immune cells (37.6 %) and PD-L1 (SP142) showed the lowest expression rate in cancer cells (11.5, 11.0, 6.9, 0.5 %) and immune cells (19.3 %) for all cut-off values (1, 5, 10, and 50 %) (Table 2; Fig. 1). The kappa value between PD-L1 (28-8) and PD-L1 (E1L3N) was higher than those between PD-L1 (28-8) and PD-L1 (SP142) and between PD-L1 (SP142) and PD-L1 (E1L3N) in both cancer cells and immune cells. Therefore, the concordance rate among monoclonal PD-L1 antibodies was higher between PD-L1 (28-8) and PD-L1 (E1L3N) (Table 3).

**Table 2:** Expression of PD-L1 monoclonal antibodies in TNBC

Parameters	PD-L1 (28-8)	PD-L1 (E1L3N)	PD-L1 (SP142)
Cancer cell compartment			
1 % cut-off value			
Negative	189 (86.7)	186 (85.3)	193 (88.5)
Positive	29 (13.3)	32 (14.7)	25 (11.5)
5 % cut-off value			
Negative	191 (87.6)	186 (85.3)	194 (89.0)
Positive	27 (12.4)	32 (14.7)	24 (11.0)
10 % cut-off value			
Negative	196 (89.9)	194 (89.0)	203 (93.1)
Positive	22 (10.1)	24 (11.0)	15 (6.9)
50 % cut-off value			
Negative	214 (98.2)	213 (97.7)	217 (99.5)
Positive	4 (1.8)	5 (2.3)	1 (0.5)
Immune cell compartment			
Negative	138 (63.3)	136 (62.4)	176 (80.7)
Low positive	59 (27.1)	55 (25.2)	40 (18.3)
High positive	21 (9.6)	27 (12.4)	2 (0.9)

**Table 3:** Kappa value for inter-PD-L1 antibodies concordance

Antibody	Tumor cell				Immune cell
	1 % cut-off	5 % cut-off	10 % cut-off	50 % cut-off	
PD-L1 (28-8) to PD-L1 (E1L3N)	0.752	0.745	0.660	0.887	0.607
PD-L1 (28-8) to PD-L1 (SP142)	0.535	0.490	0.558	0.396	0.309
PD-L1 (SP142) to PD-L1 (E1L3N)	0.537	0.551	0.580	0.328	0.305

At the 5 % cut-off value, the discordance rate between PD-L1 (28-8) and PD-L1 (E1L3N) was 6 % (13 cases) and was higher in PD-L1 (28-8)-negative/PD-L1 (E1L3N)-positive (9 cases) than PD-L1 (28-8)-positive/PD-L1 (E1L3N)-negative (4 cases) cells. The discordance rate between PD-L1 (28-8) and PD-L1 (SP142) was 10.6 % (23 cases) and was higher in PD-L1 (28-8)-positive/PD-L1 (SP142)-negative (13 cases) than PD-L1 (28-8)-negative/PD-L1 (SP142)-positive (10 cases) cells. Likewise, discordance was higher in PD-L1 (E1L3N)-positive/PD-L1 (SP142)-negative (15 cases) than PD-L1 (E1L3N)-negative/PD-L1 (SP142)-positive (7 cases) cells (Additional file 1: Table S3).

In immune cells, PD-L1 (E1L3N) had the highest and PD-L1 (SP 142) had the lowest total positive expression rate. The low-positive rate was higher with PD-L1 (28-8) than PD-L1 (E1L3N); however, the high-positive rate was higher with PD-L1 (E1L3N) than PD-L1 (28-8). In the analysis for expression concordance among the three antibodies in immune cells, the discordance rate between PD-L1 (28-8) and PD-L1 (E1L3N) was 13.8 % (30 cases), with 14 PD-L1 (28-8)-positive/PD-L1 (E1L3N)-negative cases and 16 PD-L1 (28-8)-negative/PD-L1 (E1L3N)-positive cases. The discordance rate between PD-L1 (28-8) and PD-L1 (SP142) was 24.8 % (54 cases), with 46 PD-L1 (28-8)-positive/PD-L1 (SP142)-negative cases and 8 PD-L1 (28-8)-negative/PD-L1 (SP142)-positive cases. Finally, the discordance rate between PD-L1 (E1L3N) and PD-L1 (SP142) was 22.0 % (48 cases), with 44 PD-L1 (E1L3N)-positive/PD-L1 (SP142)-negative cases and 4 PD-L1 (E1L3N)-negative/PD-L1 (SP142)-positive cases (Additional file 1: Table S4).

### Correlation of PD-L1 expression between cancer cells and immune cells

The concordance rate between cancer cells and immune cells was higher for PD-L1 (28-8) (p < 0.001). For PD-L1 (SP142), the concordance rate was higher for positive staining in cancer cells and in negative staining in immune cells (p < 0.001, Table 4).

**Table 4:** Correlation of expression of PD-L1 between cancer cell and immune cell

	PD-L1 (28-8) in cancer cell		p value
	Negative	Positive	
PD-L1 (28-8) in immune cell			<0.001
Negative	130 (68.1)	8 (29.6)	
Positive	61 (31.9)	19 (70.4)	

	PD-L1 (E1L3N) in cancer cell		p value
	Negative	Positive	
PD-L1 (E1L3N) in immune cell			0.117
Negative	120 (64.5)	16 (50.0)	
Positive	66 (35.5)	16 (50.0)	

	PD-L1 (SP142) in cancer cell		p value
	Negative	Positive	
PD-L1 (SP142) in immune cell			<0.001
Negative	163 (84.0)	13 (54.2)	
Positive	31 (16.0)	11 (45.8)	

### Correlation between PD-L1 expression and clinicopathologic factors

**Fig. 2:**
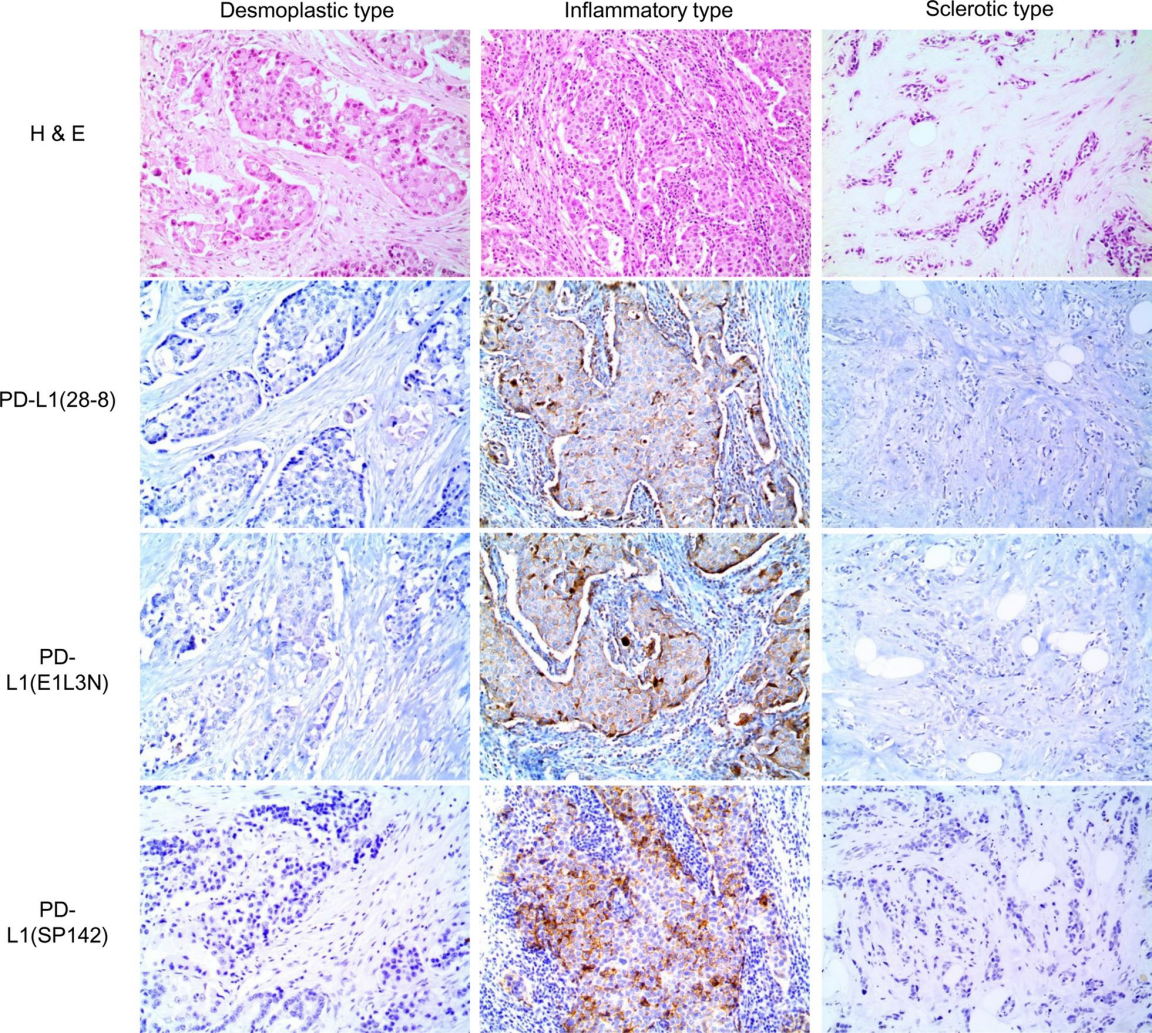
Staining with PD-L1 monoclonal antibodies according to stromal histologic type. Staining of cancer cells and surrounding immune cells using different three antibodies was higher in inflammatory type cells (Table 5)

**Fig. 3:**
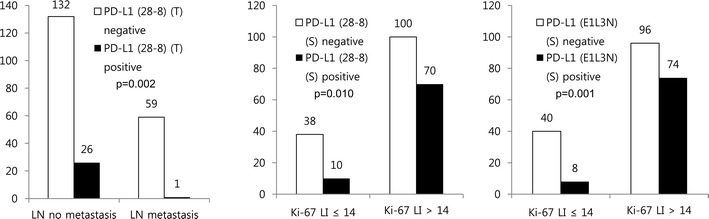
Correlation of expression of PD-L1 and clinicopathologic factors in TNBC

Correlation in cancer cells was analyzed using a 5 % cut-off value, as in most previous studies. Among the stromal histologic types, inflammatory-type cancer cells and immune cells had the highest PD-L1 expression (p < 0.05, Fig. 2). Lymph node metastasis correlated with PD-L1 (28-8) negativity in cancer cells (p = 0.002), while higher Ki-67 LI correlated with PD-L1 (28-8) positivity and PD-L1 (E1L3N) positivity in immune cells (p = 0.010 and p = 0.001, respectively) (Fig. 3).

### Impact of expression of PD-L1 on patient prognosis in TNBC

**Fig. 4:**
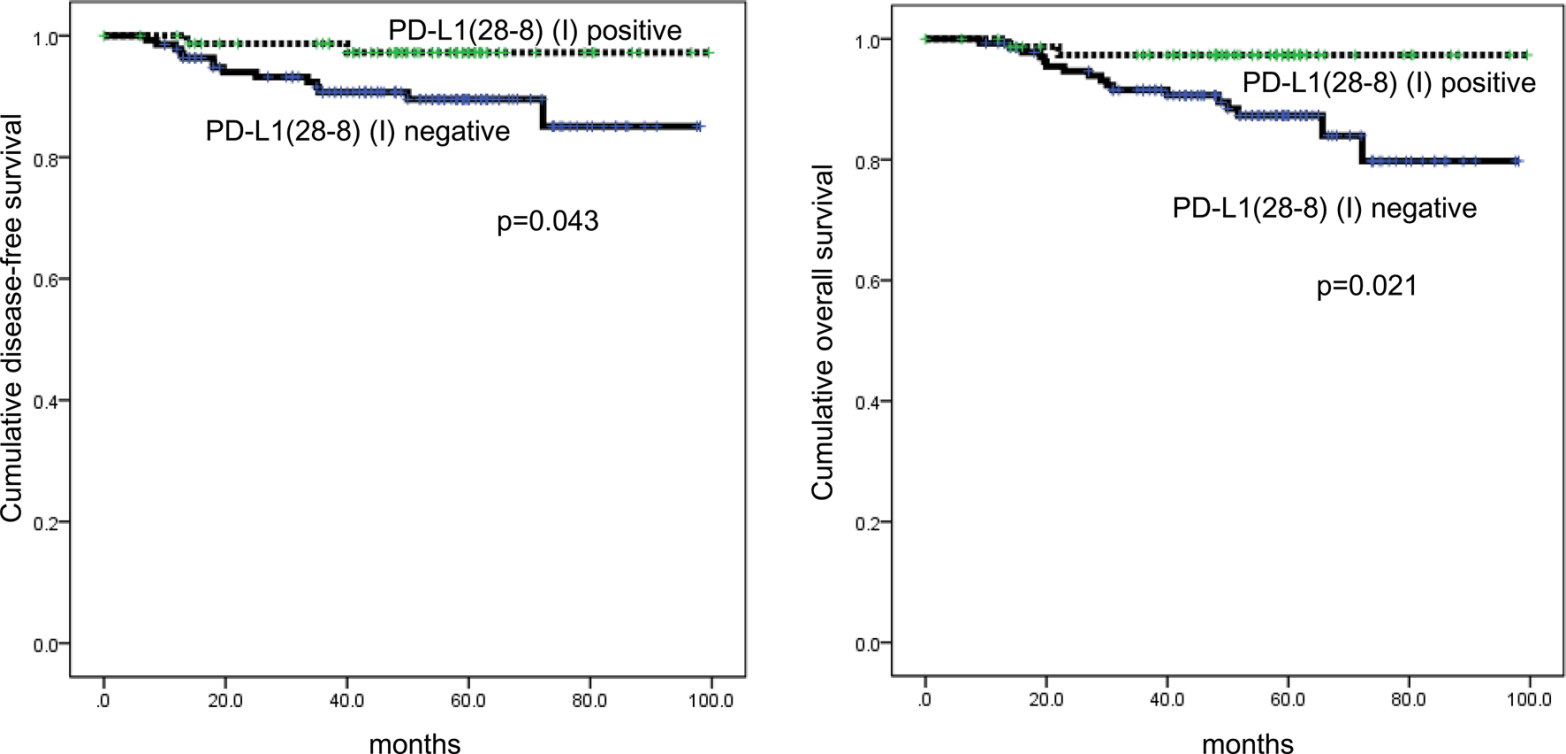
Disease-free survival and overall survival according to PD-L1 (28-8) staining in immune cells

In univariate analysis, PD-L1 (28-8) negativity in immune cells was associated with shorter disease-free survival (DFS) and overall survival (OS) (p = 0.043, and p = 0.021, Table 6; Fig. 4). In multivariate Cox analysis, factors related to shorter DFS included higher T stage (hazard ratio: 10.21, 95 % CI 1.306–79.90, p = 0.027) and lymph node metastasis (hazard ratio: 3.918, 95 % CI 1.254–12.24, p = 0.019), while factors related to shorter OS were higher T stage (hazard ratio: 6.317, 95 % CI 1.413–28.23, p = 0.016), lymph node metastasis (hazard ratio: 3.564, 95 % CI 1.304–9.740, p = 0.013), and PD-L1 (28-8) negativity in immune cells (hazard ratio: 5.112, 95 % CI 1.110–23.54, p = 0.036, Table 7).

**Table 5:** PD-L1 expression according to the stromal types in triple negative breast cancer

Parameters	Total N = 218 (%)	Stromal type			p value
		Desmoplastic n = 138 (%)	Inflammatory n = 63 (%)	Sclerotic n = 17 (%)	
PD-L1 (28-8) (T)					0.005
Negative	191 (87.6)	127 (92.0)	48 (76.2)	16 (94.1)	
Positive	27 (12.4)	11 (8.0)	15 (23.8)	1 (5.9)	
PD-L1 (E1L3N) (T)					<0.001
Negative	186 (85.3)	126 (91.3)	44 (69.8)	16 (94.1)	
Positive	32 (14.7)	12 (8.7)	19 (30.2)	1 (5.9)	
PD-L1 (SP142) (T)					0.001
Negative	194 (89.0)	130 (94.2)	48 (76.2)	16 (94.1)	
Positive	24 (11.0)	8 (5.8)	15 (23.8)	1 (5.9)	
PD-L1 (28-8) (I)					<0.001
Negative	138 (63.3)	100 (72.5)	25 (39.7)	13 (76.5)	
Positive	80 (36.7)	38 (27.5)	38 (60.3)	4 (23.5)	
PD-L1 (E1L3N) (I)					<0.001
Negative	136 (62.4)	103 (74.6)	20 (31.7)	13 (76.5)	
Positive	82 (37.6)	35 (25.4)	43 (68.3)	4 (23.5)	
PD-L1 (SP142) (I)					<0.001
Negative	176 (80.7)	121 (87.7)	40 (63.5)	15 (88.2)	
Positive	42 (19.3)	17 (12.3)	23 (36.5)	2 (11.8)	

*T* tumor cell, *I* immune cell

**Table 6:** Impact of expression of PD-L1 on disease-free and overall survival tested by log-rank analysis

Parameters	Number of patients /recurrence/death	Disease-free survival		Overall survival	
		Mean survival (95 % CI) months	p value	Mean survival (95 % CI) months	p value
PD-L1 (28-8) (T)			N/A		0.967
Negative	191/16/17	N/A		91 (87–95)	
Positive	27/0/2	N/A		59 (54–64)	
PD-L1 (E1L3N) (T)			N/A		0.647
Negative	186/16/17	N/A		90 (86–95)	
Positive	32/0/2	N/A		91 (84–98)	
PD-L1 (28-8) (I)			*0.043*		*0.021*
Negative	138/14/17	89 (84–93)		87 (82–92)	
Positive	80/2/2	97 (94–100)		97 (94–100)	
PD-L1 (E1L3N) (I)			0.761		0.255
Negative	136/11/15	91 (87–95)		88 (84–93)	
Positive	82/5/4	94 (89–98)		95 (90–99)	
PD-L1 (SP142) (I)			0.191		0.387
Negative	176/15/17	90 (86–94)		89 (85–93)	
Positive	42/1/2	97 (93–101)		95 (89–100)	

Italics represents p < 0.05

*T* tumor cell, *I* immune cell

## Discussion

This study sought to evaluate the immunohistochemical expression of PD-L1 using several monoclonal antibodies in TNBC. We performed IHC under the same conditions with different antibody clones and found differences in PD-L1 expression in both tumor cells and immune cells (Table 7).

**Table 7:** Multivariate analysis of triple negative breast cancer survival

Included parameters	Disease-free survival			Overall survival		
	Hazard ratio	95 % CI	p value	Hazard ratio	95 % CI	p value
Histologic grade			0.560			0.204
I/II versus III	0.725	0.246–2.136		0.537	0.206–1.402	
T stage			*0.027*			*0.016*
T1 versus T2/T3	10.21	1.306–79.90		6.317	1.413–28.23	
Lymph node metastasis			*0.019*			*0.013*
No versus Yes	3.918	1.254–12.24		3.564	1.304–9.740	
Ki-67 LI			0.327			0.711
≤14 versus >14	0.581	0.197–1.719		1.209	0.443–3.298	
Stromal type			0.988			0.699
Desmoplastic/sclerotic versus inflammatory	0.990	0.266–19.15		1.287	0.359–4.613	
PD-L1 (28-8) (I)			0.081			*0.036*
Negative versus positive	4.019	0.843–19.15		5.112	1.110–23.54	

Italics represents p < 0.05

*I* immune cell

The high concordance rate among the three readers in the interpretation of PD-L1 yielded acceptable inter-reader reproducibility with a kappa value >0.610 in both cancer cells and immune cells. Especially in cancer cells, the concordance rate was highest at a 1 % cut-off value and lowest at a 10 % cut-off value. This is likely because the reader tends to interpret as positive in 1 % cut-off even in slight expression and increase concordance rate, and tends to have more likely subjective interpretation in 10 % cut-off value. However, improvement in the response rate from 35 % at a 1 % cut-off value to 44 % at a 5 % cut-off value was seen in a nivolumab phase I study targeting malignant melanoma, indicating that the higher 5 % cut-off value was more reasonable [38]. Therefore, further response-based studies of TNBC are necessary.

Variation in PD-L1 expression rates previously observed in a study of various cancers has been attributed to differences in cut-off values, antibodies, and study populations [39, 40], and this phenomenon is also commonly seen with other antibodies [41]. In lung cancer characterized with PD-L1 expression, expression rates varied from 24 to 60 % using the same 5 % cut-off value [15, 16, 42], and from 21 to 95 % according to different cut-off values of 1, 10 and 50 % [13, 17, 18, 43].

PD-L1 expression in TNBC was present at 19 % (clone 5H1 with a 5 % cut-off) [44] and 64–80 % (clone E1L3N, with a 1 % cut-off) [45] in previous studies. In our study, the expression rate was 11.5–14.7 % with a 1 % cut-off value and 11.0–14.7 % with a 5 % cut-off value, which was similar to the previous report of 19 %. We presume that differences in expression rate are due primarily to different characteristics of the PD-L1 antibody clones, because all samples were stained using the same immunohistochemical conditions.

PD-L1 showed membranous expression in this study, in agreement with one previous study in TNBC [44], but the other TNBC study reported both cytoplasmic and membranous expression of PD-L1 [45]. PD-L1 expression has been reported in the cell membrane [17, 26–28] or membrane and cytoplasm [13, 16, 18] in other cancers. We performed IHC using an automatic IHC staining device, but the previous research in TNBC that evaluated cytoplasm expression had no clear description of staining, making it difficult to compare our findings directly with previous data [45]. We found expression of PD-L1 in 19.3–37.6 % of cancer cells and immune cells, depending on the antibody clone. Although our expression rate was different than a previous study with 93 % immune cell expression in TNBC, it was in concordance with the previous report in terms of positive immune cell expression [45]. PD-L1 expression in immune cells has been reported in other cancers [16, 19, 25, 28]. There are differences between oncogene-driven PD-L1 expression and inflammation-driven PD-L1 expression. While oncogene-driven PD-L1 expression is constitutive and diffuse, inflammation-driven PD-L1 expression is limited to sites of IFNγ-mediated immunologic attack [46]. In addition, inflammation-driven PD-L1 expression is related to immune infiltrates, while oncogene-driven PD-L1 expression is not [28, 47]. Thus, further study to know which expression between two expressions is more related to the expression of PD-L1 on immune cell in TNBC.

In this study, the concordance rate between PD-L1 (28-8) and PD-L1 (E1L3N) was high in both cancer cells and immune cells while PD-L1 (SP142) showed low concordance rates with the other two antibodies. Previous studies reported poor concordance (kappa value: 0.124–0.340) between PD-L1 (E1L3N) and PD-L1 (SP142) in lung cancer [48], but a high concordance rate between PD-L1 (E1L3N) and PD-L1 (SP142) (more than 85 %) in malignant melanoma [49]. Therefore, concordance seems to vary according to cancer type; further study on this topic is needed.

PD-L1 positivity in immune cells (28-8 clone) was an independent prognostic factor in our study, in contrast to previous studies showing good prognosis [50–53] or poor prognosis [54, 55] with PD-L1 expression in cancer cells. Since the prognostic and predictive significance of PD-L1 expression in immune cells such as macrophages and lymphocytes was not associated with tumor-related PD-L1 expression [16], further study of the biologic implications of PD-L1 in immune cells during TNBC is needed.

In breast cancer, tumor infiltrating lymphocytes (TILs) have been linked with good prognosis [56], as have stromal TIL [57]. Therefore, tumor immunity may relate to prognosis in TNBC. PD-L1 expression was significantly higher in inflammatory type cancer cells and immune cells with all tested antibodies. Therefore, we assume that PD-L1 expression in stromal immune cells is related to prognosis, and further research is needed to better elucidate this relationship.

Clinical trials targeting PD-L1 are underway [17, 58–60] and good responses have been reported [61, 62]. In TNBC, which has no effective therapy, PD-L1-targeting agents may play an important role and strong biomarkers that can predict treatment response are needed. Accordingly, staining of PD-L1 monoclonal antibodies may act as a biomarker for PD-L1-targeting agents, but clinical trials evaluating TNBC response to PD-L1-targeting agents using monoclonal antibody staining will be needed to validate this strategy.

The limitation of this study was the potential difference in results between TMA and the whole cancer tissue section. Breast cancer also shows intracancer heterogeneity, like other types of cancer, and the expression of PD-L1 may cause differences between TMA and the whole cancer tissue section. PD-L1 expression in lung cancer showed a high discordance rate between TMA samples and whole tissue sections [48]. In a previous study, the results of immunohistochemical staining of ER in breast cancer were different in 5.5 % between TMA and the whole cancer tissue section. Furthermore, when the number of cores was greater than one, this difference decreased to 1.4 % [63]. In this study, two core extractions per case were performed to reduce this bias.

## Conclusions

In conclusion, staining with PD-L1 (clone 28-8), PD-L1 (clone E1L3N), and PD-L1 (clone SP142) monoclonal antibodies in cancer cells and immune cells varied, with the highest staining by PD-L1 (E1L3N) and the lowest by PD-L1 (SP142). The concordance rate among monoclonal PD-L1 antibodies was higher between PD-L1 (28-8) and PD-L1 (E1L3N). PD-L1 positivity in immune cells correlated with a favorable prognosis. To determine the gold standard antibody and the most appropriate cut-off value, further study of the clinical trial group treated with PD-L1 inhibitor is necessary.

## Additional file

10.1186/s12967-016-0925-6Additional Tables, Tables S1–S4.

## Abbreviations

TNBC: triple-negative breast cancer
ER: estrogen receptor
PR: progesterone receptor
HER2: human epidermal growth factor receptor 2
PD: programmed death
PD-L: programmed death ligand
IHC: immunohistochemistry
FFPE: formalin-fixed and paraffin-embedded
NOS: not otherwise specified
ASCO: American Society of Clinical Oncology
CAP: College of American Pathologists
H&E: hematoxylin and eosin
AJCC: American Joint Committee on Cancer
DFS: disease-free survival
OS: overall survival
HPF: high power field
