# Supplementary material for: Expression of PD-L1 in triple-negative breast cancer based on different immunohistochemical antibodies
Source: J Transl Med. 2016 Jun 10;14:173. doi: 10.1186/s12967-016-0925-6 (PMC4902914; doi:10.1186/s12967-016-0925-6)
Supplement: Supplementary file 1 — 10.1186/s12967-016-0925-6Additional Tables, Tables S1–S4. [file 12967_2016_925_MOESM1_ESM.doc]

| **Supplementary Table 1:** Source, clone, and dilution of the antibodies used | | | | | | | |  |
| --- | --- | --- | --- | --- | --- | --- | --- | --- |
| **Antibody** | **Company** | **Immunogen** | **Application** | **Clone** | **Antigen retrieval** | **Positive control** | **Dilution** | |
| PD-L1 | Abcam, Cambridge, UK | extracellular domain of huPD-L1 (Phe19-Thr239) | IHC-P, WB, Flow cytometry | 28-8 | CC1 | Placenta, tonsil | 1:100 |  |
| PD-L1 | Spring Biosciences,  USA, Fremont, CA | C-terminus of human PD-L1 protein | IHC-P | SP142 | CC1 | Placenta, tonsil | 1:100 |  |
| PD-L1 | Cell Signaling Technology,  USA, Beverly, MA | C-terminus of human PD-L1 protein | IHC-P, WB, Flow cytometry, Immunoprecipitation | E1L3N | CC1 | Placenta, tonsil | 1:100 |  |

CC1, Cell Conditioning 1

| **Supplementary Table 2.** Basal characteristics of triple negative breast cancer according to the stromal types | | | | | |
| --- | --- | --- | --- | --- | --- |
| Parameters | Total  N=218 (%) | Stromal type | | | p-value |
|  |  | Desmoplastic  n=138 (%) | Inflammatory  n=63 (%) | Sclerotic  n=17 (%) |  |
| Age (year) |  |  |  |  | 0.988 |
| ≤ 35 | 28 (12.8) | 18 (13.0) | 8 (12.7) | 2 (11.8) |  |
| > 35 | 190 (87.2) | 120 (87.0) | 55 (87.3) | 15 (88.2) |  |
| Histologic grade |  |  |  |  | 0.562 |
| I | 5 (2.3) | 2 (1.4) | 3 (4.8) | 0 (0.0) |  |
| II | 51 (23.4) | 33 (23.9) | 15 (23.8) | 3 (17.6) |  |
| III | 162 (74.3) | 103 (74.6) | 45 (71.4) | 14 (82.4) |  |
| T stage |  |  |  |  | 0.227 |
| 1 | 99 (45.4) | 65 (47.1) | 27 (42.9) | 7 (41.2) |  |
| 2 | 117 (53.7) | 72 (52.2) | 36 (57.1) | 9 (52.9) |  |
| 3 | 2 (0.9) | 1 (0.7) | 0 (0.0) | 1 (5.9) |  |
| Lymph node metastasis |  |  |  |  | 0.610 |
| No | 158 (72.5) | 99 (71.7) | 48 (76.2) | 11 (64.7) |  |
| Yes | 60 (27.5) | 39 (28.3) | 15 (23.8) | 6 (35.3) |  |
| Ki-67 LI |  |  |  |  | 0.330 |
| ≤ 14 | 48 (22.0) | 33 (23.9) | 10 (15.9) | 5 (29.4) |  |
| > 14 | 170 (78.0) | 105 (76.1) | 53 (84.1) | 12 (70.6) |  |
| Tumor recurrence | 16 (7.3) | 11 (8.0) | 3 (4.8) | 2 (11.8) | 0.553 |
| Patients death | 19 (8.7) | 13 (9.4) | 3 (4.8) | 3 (17.6) | 0.220 |

| **Supplementary Table 3.** Comparison of Expression of PD-L1 monoclonal antibodies in cancer cells of TNBC by 5% cut-off value | | | | |
| --- | --- | --- | --- | --- |
| Parameters | PD-L1 (E1L3N) | | PD-L1 (SP142) | |
|  | Negative | Positive | Negative | Positive |
| PD-L1 (28-8) |  |  |  |  |
| Negative | 182 (97.8) | 9 (28.1) | 181 (93.3) | 10 (41.7) |
| Positive | 4 (2.2) | 23 (71.9) | 13 (6.7) | 14 (58.3) |
| PD-L1 (SP142) |  |  |  |  |
| Negative | 179 (92.3) | 15 (7.7) | N/A | N/A |
| Positive | 7 (29.2) | 17 (70.8) | N/A | N/A |

| **Supplementary Table 4.** Comparison of expression of PD-L1 monoclonal antibody in immune cells of TNBC | | | | | | |
| --- | --- | --- | --- | --- | --- | --- |
| Parameters | PD-L1 (E1L3N) | | | PD-L1 (SP142) | | |
|  | Negative | Low positive | High positive | Negative | Low positive | High positive |
| PD-L1 (28-8) |  |  |  |  |  |  |
| Negative | 122 (89.7) | 16 (29.1) | 0 (0.0) | 130 (73.9) | 8 (20.0) | 0 (0.0) |
| Low positive | 13 (9.6) | 35 (63.6) | 11 (40.7) | 39 (22.2) | 20 (50.0) | 0 (0.0) |
| High positive | 1 (0.7) | 4 (7.3) | 16 (59.3) | 7 (4.0) | 12 (30.0) | 2 (100.0) |
| PD-L1 (SP142) |  |  |  |  |  |  |
| Negative | 132 (97.1) | 39 (70.9) | 5 (18.5) | N/A | N/A | N/A |
| Low positive | 4 (2.9) | 16 (29.1) | 20 (74.1) | N/A | N/A | N/A |
| High positive | 0 (0.0) | 0 (0.0) | 2 (7.4) | N/A | N/A | N/A |
